# Comparison of adverse events, prescription medication, and costs after hip, knee, and shoulder total joint arthroplasty: a retrospective cohort study

**DOI:** 10.1186/s42836-025-00309-y

**Published:** 2025-05-06

**Authors:** Maggie E. Horn, Steven Z. George, Anna Giczewska, Brooke Alhanti, Irene L. Tanner, Michael P. Bolognesi

**Affiliations:** 1 Department of Orthopaedic Surgery, Duke University, Durham, NC 27710 USA; 2 Department of Population Health Sciences, Duke University, Durham, NC 27701 USA; 3 Duke Clinical Research Institute, Duke University, Durham, NC 27701 USA; 4 Department of Biostatistics and Bioinformatics, Duke University, Durham, NC 27710 USA

**Keywords:** Adverse events, Complications, Cost, Osteoarthritis, Total joint arthroplasty

## Abstract

**Background:**

Outcomes from Total Joint Arthroplasty (TJA) are variable but generally favorable. However, the literature is lacking regarding direct comparisons of important outcomes across TJA sites. Such comparisons are of paramount importance to informing future bundled care reform and patient optimization. Thus, we compared the rates of adverse events, filled prescriptions, and costs at 90 days and 365 days after TJA for knee, hip, and shoulder patients.

**Methods:**

We conducted a retrospective cohort study of multi-payor claims data with patients (*n* = 2416) who underwent hip (*n* = 909), knee (*n* = 1250), or shoulder (*n* = 257) TJA within an academic health system. Univariable and multivariable logistic regression models were used to assess the association between the TJA surgical site and adverse events (i.e., medical and surgical complications) and prescriptions filled. Univariable and multivariable gamma regression models were used to assess the association between the TJA surgical site and total cost and surgical episode cost.

**Results:**

In all regression models, the hip location was used as the reference group. There were no differences in the adjusted odds of medical complications between the TJA surgical sites after adjusting for confounders at 90 days or 365 days. For surgical complications, the adjusted odds were 2.66 times higher in the knee (*P* < 0.001) and 4.48 times higher in the shoulder (*P* < 0.001) at 90 days. At 365 days, the odds were 2.54 times higher in the knee (*P* < 0.001) and 4.10 times higher in the shoulder (*P* < 0.001). There was an increase in the adjusted odds of antiepileptic and NSAIDS being filled in knee and shoulder patients compared to hip patients at 31–90 days (both *P* < 0.001). At 0–365 days, knee patients had increased adjusted odds of filled antibiotic (*P* = 0.032), antiepileptic (*P* = 0.001), and opioid (*P* = 0.005) prescriptions compared to hip patients, while shoulder patients only increased odds of antiepileptic (*P* = 0.028). Lastly, in adjusted models, both the knee and shoulder had a significant increase in total health system costs, with a 9% and 14% increase in cost, respectively (*P* < 0.01).

**Conclusion:**

Patients undergoing TKA and TSA may have an increased risk for surgical complications and longer-term opioid prescriptions (TKA only) compared to those undergoing THA. Collectively, these results can inform future population-based approaches to managing osteoarthritis care pathways or reimbursement policies for TJA across multiple joint sites.

**Supplementary Information:**

The online version contains supplementary material available at 10.1186/s42836-025-00309-y.

## Introduction

Total joint arthroplasty (TJA) is generally believed to be an effective treatment for severe hip, knee, and shoulder osteoarthritis [1–3]. Accordingly, total hip arthroplasty (THA) is the most frequently performed joint replacement surgery worldwide, with an estimated 400,000 procedures performed annually in the United States alone [4]. Similarly, total knee arthroplasty (TKA) and total shoulder arthroplasty (TSA) are also commonly performed, with an estimated 790,000 [5] and 53,000 [6] procedures performed annually in the United States, respectively. While outcomes from TJA are generally favorable, some variation is noted at the surgical site. For example, TKA has higher resource utilization (e.g., increased hospital days and physical therapy visits) when compared to THA and TSA [7]. However, the literature is lacking regarding the comparison of important outcomes like adverse events, medication use, and costs across TJA sites.

The relevance of considering such outcomes is linked to the development of population-based management for musculoskeletal conditions [8]. In the United States and countries with single-payer healthcare systems, value-based care initiatives for managing THA and TKA patients have paved the way for population-based management of individuals with osteoarthritis by collectively considering conservative and surgical options [9]. In the United States, since the voluntary initiation of Bundle Payments for Care Improvement and Comprehensive Care for Joint Replacement (BCPI) [10] and mandatory Comprehensive Care for Joint Replacement (CCJR) [11] bundle participation, TKA and THA surgeons have had a significant change in their approach to patients with end-stage osteoarthritis [12]. Similarly, in countries with single-payer healthcare systems, financial systems affect wait times, how patients are evaluated and screened before surgery, as well as the criteria required to be deemed indicated for elective TKA and THA [13]. However, to date, there has not been an effort to explore patient optimization and value-based care for an emergent procedure like TSA along with established procedures like TKA and THA [14, 15]. This approach is innovative as these surgeries are often considered separately, which is inconsistent with a population-based understanding of the impact of TJA as a management option for osteoarthritis.

Therefore, in this retrospective cohort study, we compare patients undergoing TKA, THA, and TSA using retrospective claims data from two payors. The primary aim of this study was to compare the rates of adverse events (medical or surgical complications) at 90 days and 365 days after TJA for these three sites. The secondary aim was to compare the rates of filled prescriptions at 31–90 days and 365 days after TJA. Lastly, we compared the relative difference in costs of TJA for these three sites.

## Methods

### Study design

We conducted a retrospective cohort study of patients who underwent TJA within an academic health system. This study was approved, and it was determined that the requirement for informed consent was waived. This study follows the Strengthening the Reporting of Observational Studies in Epidemiology (STROBE) reporting guideline for observational studies [16].

### Data source and population

**Fig. 1:**
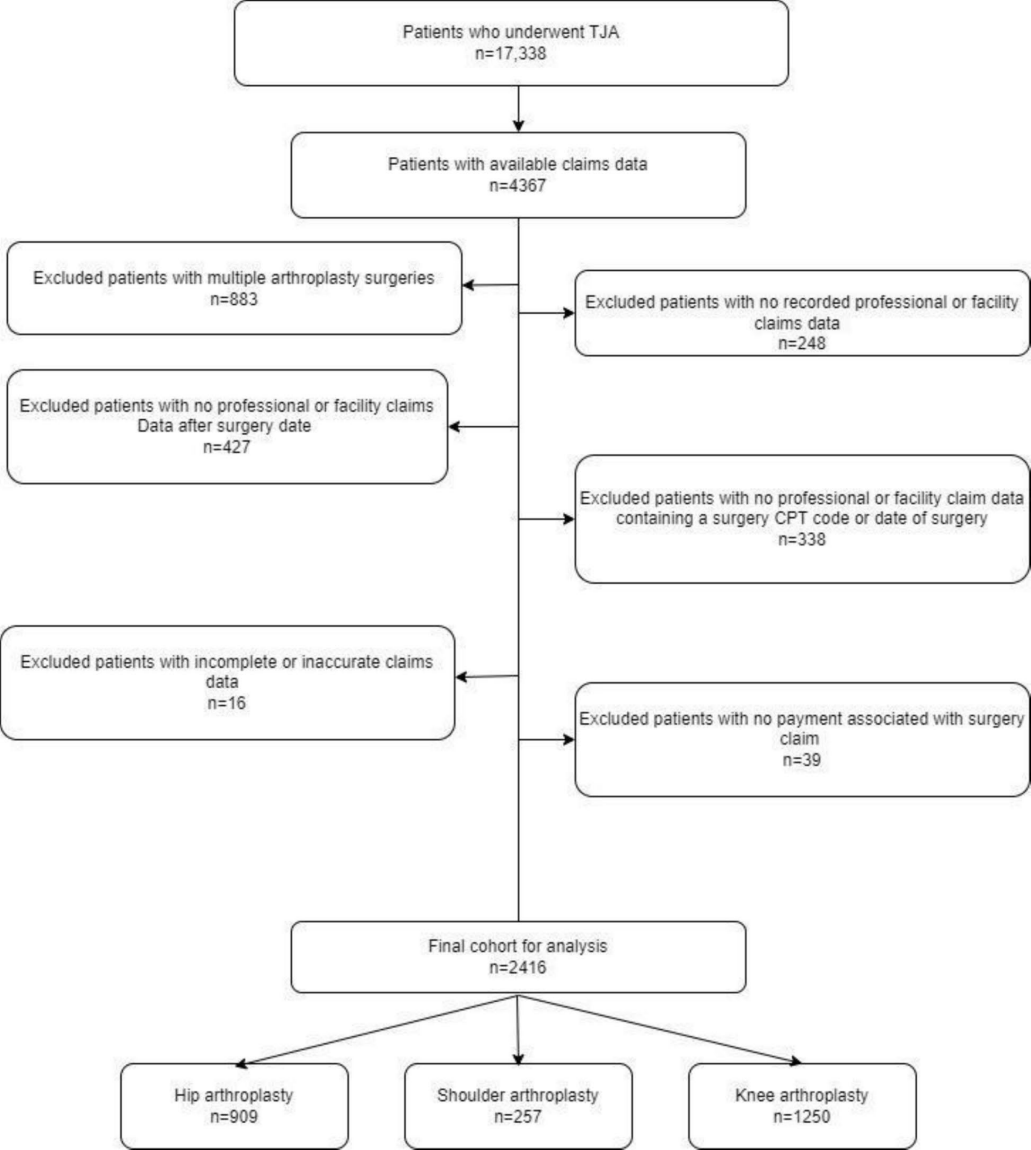
The derivation of the cohort with inclusion and exclusion criteria applied to arrive at the final analytical dataset

We identified a cohort of patients from the electronic health record (EHR) who underwent hip, knee, or shoulder TJA from January 1, 2014, through February 1, 2020. From this cohort, we queried an internal claims database for two payors (private insurer and Medicare) to identify patients with complete claims data (Facilities and Pharmacy) 365 days before and after the arthroplasty procedure. The inclusion criteria for this study were patients 18 years and older who underwent TKA, THA, or TSA procedures. Patients were excluded from the analysis if they did not have associated claims data, had multiple arthroplasty procedures, or if claims data were missing or inaccurate for the surgical event. We did not apply any further exclusion criteria based on clinical characteristics or surgical indications (i.e., osteoarthritis, arthrosis, or fracture) Fig. 1.

### Measures

Our primary outcomes were medical and surgical adverse events among patients who underwent THA, TKA, or TSA. Our secondary outcomes were medications filled (opioids, antibiotics, NSAIDs, and antiepileptics) and costs among patients who had THA, TKA, and TSA procedures. First, we categorized adverse events into death, medical, and surgical complications using ICD-9 and ICD-10 codes [17]. We recorded the cumulative rates of adverse events within 90 days and 365 days from the day of discharge date. Death was recorded using the death field in the claims database. Medical Complications were recorded if there was a CPT code billed for thromboembolic disease, osteolysis, Myocardial Infarction, heart failure, respiratory failure, stroke, acute renal function, or urinary tract infection. Surgical complications were recorded if there was a CPT code billed for wound complications, bleeding, neural deficit, vascular injury, instability, stiffness, periprosthetic fracture, extensor mechanism disruption, bearing surface wear, implant loosening, or deep periprosthetic joint infection (Appendix 1).

Our second aim was to examine the rates of filled prescriptions at 31–90 days and 0–365 days after TJA. The period from discharge to 365 days reflects overall prescription rates, and the 31–90-day period reflects the sub-acute postoperative period. We categorized filled prescriptions into the categories of antibiotics, antiepileptics, non-steroidal anti-inflammatories (NSAIDs), and opioids using the National Drug Code Directory [18]. We recorded the percentage of patients who were prescribed at least one of the medications and who filled the prescription within 31–90 days and 0–365 days of discharge. Lastly, we compared the relative costs between TJA groups. We calculated the surgical episode cost as defined by the total payment related to the surgery and post-operative hospital stay. Next, we calculated the total healthcare costs and surgical episode costs as defined by total payments for all claims from the date of the surgery claim to 365 days. We converted costs to adjust for inflation [19]. 

A priori, we calculated two covariates to be included in the regression models: The Elixhauser Comorbidity Index (ECI) and the Opioid Morphine Milligram Equivalent (MME). The ECI is a measure of comorbidity used in epidemiology and clinical research [20]. It is a set of 30 clinically relevant conditions that are commonly found among hospitalized patients. The index is calculated by counting the number of conditions present in a patient's medical history and assigning a score of 0 (no comorbidity) to 30 (maximum comorbidity). The ECI is commonly used in research studies to control for the effects of comorbidity on outcomes. We reported the frequency of comorbidities, ECI readmission score, and ECI morbidity index [21]. To control for opioid use on outcomes, we calculated the average daily MME of opioids within the time window of the endpoint of interest (90 days and 365 days). The MME was calculated by identifying opioid prescriptions using medication names (i.e., Propoxyphene, Codeine, Hydrocodone, Tramadol, Dihydrocodeine, Pentazocine, Morphine, Oxycodone, Hydromorphone, Meperidine hydrochloride, Oxymorphone, Levorphanol, Methadone, Fentanyl, Buprenorphine, Opium, Tapentadol). For each opioid prescription, we calculated the daily MME as Morphine equivalent conversion factor (mg morphine/mg) × strength (mg/unit) × metric quantity (unit)/days supplied (day). The average daily MME was then calculated by multiplying the daily MME by the number of days this prescription was active.

### Statistical analysis

Cohort characteristics were reported overall and by the TJA group. Counts and percentages were calculated for categorical variables, and the median and 25 th and 75 th percentiles were calculated for continuous variables. Adverse events (surgical and medical complications) were summarized as the frequency and percentage of patients who experienced the adverse event within 90 days and 365 days of the surgical event.

Univariable and multivariable logistic regression models were performed to assess the association between the TJA group and specific adverse events of medical and surgical complications at 90 and 365 days. We also performed univariable and multivariable logistic regression models to assess the association of opioid prescription at 31–90 days and 0–365 days. Univariable and multivariable Gamma regression models were used to assess the association between the TJA group and cost-of-care, including total cost and surgical episode cost. Effect estimates or odds ratios (OR), 95% confidence intervals (CI), and associated p-values are presented. THA was used as the reference group in all models. Two statisticians (AG, BA) designed and independently carried out the statistical analysis for this study.

## Results

A sample of 2416 patients underwent TJA procedures and met our criteria for being analyzed, including TKA (*n* = 1250), THA (*n* = 909), and TSA (*n* = 257) patients. The median age of the cohort was 70 years old (25 th: 66, 75 th: 76), and the majority of the cohort was female (60.3%) and Caucasian/White (79.9%) (Table 1).

**Table 1:** Patient demographics

		**TJA groups**		
	**Overall** ***n* = 2416**	**Knee** ***n* = 1250**	**Hip** ***n* = 909**	**Shoulder** ***n* = 257**
**Baseline characteristics**				
**Age** [median (25 th, 75 th)]	70 (66–75)	70 (67–75)	70 (65–75)	71 (67–76)
**Sex**				
Female	1458/2416 (60.3%)	783/1250 (62.6%)	524/909 (57.6%)	151/257 (58.8%)
Male	958/2416 (39.7%)	467/1250 (37.4%)	385/909 (42.4%)	106/257 (41.2%)
**Race**				
American Indian or Alaskan Native	< 11/2416 (< 0.5%)	< 11/1250 (< 0.9%)	< 11/909 (< 1.2%)	0/257 (0.00%)
Asian	> 24/2416 (> 0.1%)	22/1250 (1.80%)	< 11/909 (< 1.2%)	< 11/257 (< 4.3%)
Black or African American	413/2416 (17.1%)	229/1250 (18.3%)	149/909 (16.4%)	35/257 (13.6%)
Caucasian/White	1930/2416 (79.9%)	972/1250 (77.8%)	739/909 (81.3%)	219/257 (85.2%)
Not reported	< 33/2416 (< 1.4%)	< 11/1250 (< 0.9%)	< 11/909 (< 1.2%)	< 11/257 (< 4.3%)
Other	44/2416 (1.0%)	18/1250 (1.4%)	< 11/909 (< 1.2%)	< 11/257 (< 4.3%)
**Ethnicity**				
Hispanic or Latino	< 30/2416 (< 1.2%)	19/1250 (2.5%)	< 11/909 (< 1.2%)	< 11/257 (< 4.3%)
Not Hispanic/Latino	2355/2416 (97.5%)	1219/1250 97.5%)	885/909 (97.4%)	251/257 (97.7%)
Not reported	> 30/2416 (> 1.2%)	12/1250 (1.0%)	> 18/909 (> 2.0%)	< 11/257 (< 4.3%)
**BMI** [median (25 th, 75 th)]	29.57 (25.97–33.59)	30.65 (26.98–34.64)	27.98 (24.84–32.28)	29.41 (26.05–33.50)
**Tobacco use**				
Never	1177/2401 (49.0%)	626/1244 (50.3%)	424/902 (47.0%)	127/255 (49.8%)
Passive	< 11/2401 (0.5%)	< 11/1244 (< 0.9%)	< 11/902 (< 1.2%)	0/255 (0.00%)
Quit	> 1070/2401 (> 44.6%)	> 550/1244 (> 44.2%)	> 48/902 (> 45.2%)	112/255 (43.9%)
Yes	143/2401 (6.0%)	61/1244 (4.9%)	66/902 (7.3%)	16/255 (6.3%)
**Alcohol**				
Defer	< 11/2399 (< 0.5%)	< 11/1244 (< 0.9%)	< 11/901 (< 1.2%)	0/254 (0.00%)
Never	> 27/2399 (> 1.1%)	15/1244 (1.2%)	11/901 (1.2%)	< 11/254 (< 4.3%)
No	1011/2399 (42.1%)	550/1244 (44.2%)	356/901 (39.5%)	105/254 (41.3%)
Not asked	< 11/2399 (< 0.5%)	< 11/1244 (< 0.9%)	< 11/901 (< 1.2%)	< 11/254 (< 4.3%)
Not currently	61/2399 (2.5%)	19/1244 (1.5%)	29/901 (3.2%)	13/254 (5.1%)
Yes	1291/2399 (53.8%)	659/1244 (53.0%)	501/901 (55.6%)	131/254 (51.6%)
**Elixhauser comorbidities**				
AIDS	< 22/2416 (< 0.9%)	< 11/1250 (< 0.9%)	< 11/909 (< 1.2%)	0/257 (0.0%)
Alcohol abuse^a^	< 22/2416 (< 0.9%)	< 11/1250 (< 0.9%)	< 11/909 (< 1.2%)	0/257 (0.0%)
Deficiency Anemias	422/2416 (17.5%)	230/1250 (18.4%)	134/909 (14.7%)	58/257 (22.6%)
Rheumatoid arthritis/collagen vas	181/2416 (7.5%)	95/1250 (7.6%)	65/909 (7.2%)	21/257 (8.2%)
Chronic blood loss anemia	> 36/2416 (> 1.5%)	18/1250 (1.4%)	17/909 (1.9%)	< 11/257 (< 4.3%)
Congestive heart failure	168/2416 (7.0%)	76/1250 (6.1%)	65/909 (7.2%)	27/257 (10.5%)
Chronic pulmonary disease	413/2416 (17.1%)	215/1250 (17.2%)	146/909 (16.1%)	52/257 (20.2%)
Coagulopathy	99/2416 (4.1%)	53/1250 (4.2%)	34/909 (3.7%)	12/257 (4.7%)
Depression^a^	434/2416 (18.0%)	221/1250 (17.7%)	147/909 (16.2%)	66/257 (25.7%)
Diabetes w/o chronic complications	501/2416 (20.7%)	284/1250 (22.7%)	150/909 (16.5%)	67/257 (26.1%)
Diabetes w/chronic complications	249/2416 (10.3%)	139/1250 (11.1%)	75/909 (8.3%)	35/257 (13.6%)
Drug abuse^a^	0/2416 (0.0%)	0/1250 (0.0%)	0/909 (0.0%)	0/257 (0.0%)
Hypertension	1716/2416 (71.0%)	923/1250 (73.8%)	610/909 (67.1%)	183/257 (71.2%)
Hypothyroidism	447/2416 (18.5%)	247/1250 (19.8%)	154/909 (16.9%)	46/257 (17.9%)
Liver disease	111/2416 (4.6%)	56/1250 (4.5%)	39/909 (4.3%)	16/257 (6.2%)
Lymphoma	> 22/2416 (> 0.9%)	12/1250 (1.0%)	< 11/909 (< 1.2%)	< 11/257 (< 4.3%)
Fluid and electrolyte disorders	338/2416 (14.0%)	161/1250 (12.9%)	137/909 (15.1%)	40/257 (15.6%)
Metastatic cancer	> 37/2416 (> 1.5%)	13/1250 (1.0%)	18/909 (2.0%)	< 11/257 (< 4.3%)
Other neurological disorders	218/2416 (9.0%)	110/1250 (8.8%)	78/909 (8.6%)	30/257 (11.7%)
Obesity	540/2416 (22.4%)	307/1250 (24.6%)	168/909 (18.5%)	65/257 (25.3%)
Paralysis	< 33/2416 (< 1.4%)	< 11/1250 (< 0.9%)	< 11/909 (< 1.2%)	< 11/257 (< 4.3%)
Peripheral vascular disease	273/2416 (11.3%)	125/1250 (10.0%)	113/909 (12.4%)	35/257 (13.6%)
Psychoses^a^	197/2416 (8.2%)	103/1250 (8.2%)	62/909 (6.8%)	32/257 (12.5%)
Pulmonary circulation disease	> 80/2416 (> 3.3%)	36/1250 (2.9%)	37/909 (4.1%)	< 11/257 (< 4.3%)
Renal failure	195/2416 (8.1%)	84/1250 (6.7%)	82/909 (9.0%)	29/257 (11.3%)
Solid tumor w/out metastasis	363/2416 (15.0%)	186/1250 (14.9%)	136/909 (15.0%)	41/257 (16.0%)
Peptic ulcer disease without bleeding	> 20/2416 (> 0.8%)	10/1250 (0.8%)	10/909 (1.1%)	< 11/257 (< 4.3%)
Valvular disease	277/2416 (11.5%)	140/1250 (11.2%)	103/909 (11.3%)	34/257 (13.2%)
Weight loss	> 68/2416 (> 2.8%)	29/1250 (2.3%)	33/909 (3.6%)	< 11/257 (< 4.3%)
**Elixhauser calculated scores**				
Mortality Index Score [median (25 th, 75 th)]	0 (− 1–6)	0 (− 2–6)	0 (− 1–7)	0 (− 2–7)
Readmission Index Score [median (25 th, 75 th)]	9 (0–21)	10 (0–21)	8 (0–20)	11 (2–28)

^a^Mental health and substance abuse claims were withheld from the Medicare claims

### Adverse events

Medical complications.

For all-cause medical complications, at 90 days, the rate of overall complications across all groups was 14.4%, and at 365 days, it was 24.1%. The highest rate of complications at 365 days was found in the shoulder group (32.3%) compared to the knee (23.8%) and hip (22.3%) groups. Urinary tract infection was the most frequent medical complication at 90 days (6.0%) and 365 days (11.9%). Table 2. There was no significant difference in the adjusted odds of complications in knee and shoulder TJA groups compared to the hip after adjusting for confounders at 90 days (knee: *P* = 0.427; shoulder *P* = 0.650) and 365 days (knee: *P* = 0.878; shoulder: *P* = 0.186) (Table 3).

**Table 2:** Adverse events

	**Overall** ***n* = 2416**	**Knee** ***n* = 1250**	**Hip** ***n* = 909**	**Shoulder** ***n* = 257**
**Death**				
90 days	0/2416 (0.00%)	0/1250 (0.00%)	0/909 (0.00%)	0/257 (0.00%)
365 days	< 11/2416 (< 0.5%)	< 11/1250 (< 0.9%)	0/909 (0.00%)	0/257 (0.00%)
**All cause medical complications**				
90 days	348/2416 (14.40%)	174/1250 (13.90%)	130/909 (14.30%)	44/257 (17.10%)
365 days	583/2416 (24.10%)	297/1250 (23.80%)	203/909 (22.30%)	83/257 (32.30%)
Thromboembolic disease				
90 days	> 43/2416 (> 1.8%)	23/1250 (1.8%)	17/909 (1.9%)	< 11/257 (< 4.28%)
365 days	> 74/2416 (> 3.0%)	38/1250 (3.0%)	28/909 (3.1%)	< 11/257 (< 4.28%)
Osteolysis				
90 days	0/2416 (0.0%)	0/1250 (0.0%)	0/909 (0.0%)	0/257 (0.0%)
365 days	0/2416 (0.0%)	0/1250 (0.0%)	0/909 (0.0%)	0/257 (0.0%)
Myocardial infarction				
90 days	< 33/2416 (< 1.4%)	< 11/1250 (< 0.9%)	< 11/909 (< 1.2%)	< 11/257 (< 4.28%)
365 days	> 30/2416 (> 1.2%)	14/1250 (1.1%)	15/909 (1.7%)	< 11/257 (< 4.28%)
Heart failure				
90 days	119/2416 (4.9%)	56/1250 (4.5%)	51/909 (5.6%)	12/257 (4.7%)
365 days	171/2416 (7.1%)	75/1250 (6.0%)	68/909 (7.5%)	28/257 (10.9%)
Respiratory failure				
90 days	32/2416 (1.3%)	13/1250 (1.0%)	< 11/909 (< 1.2%)	< 11/257 (< 4.28%)
365 days	66/2416 (2.7%)	33/1250 (2.6%)	20/909 (2.2%)	13/257 (5.1%)
Stroke				
90 days	> 30/2416 (> 1.2%)	12/1250 (1.0%)	13/909 (1.4%)	< 11/257 (< 4.28%)
365 days	84/2416 (3.5%)	43/1250 (3.4%)	26/909 (2.9%)	15/257 (5.8%)
Acute renal function				
90 days	> 41/2416 (> 1.7%)	27/1250 (2.2%)	12/909 (1.3%)	< 11/257 (< 4.28%)
365 days	110/2416 (4.6%)	60/1250 (4.8%)	36/909 (4.0%)	14/257 (5.4%)
Urinary tract infection				
90 days	145/2416 (6.0%)	75/1250 (6.0%)	53/909 (5.8%)	17/257 (6.6%)
365 days	287/2416 (11.9%)	150/1250 (12.0%)	103/909 (11.3%)	34/257 (13.2%)
**All cause surgical complications**				
90 days	480/2416 (19.9%)	307/1250 (24.6%)	87/909 (9.6%)	86/257 (33.5%)
365 days	513/2416 (21.2%)	323/1250 (25.8%)	100/909 (11.0%)	90/257 (35.0%)
Wound complications				
90 days	< 22/2416 (< 0.9%)	< 11/1250 (< 0.9%)	< 11/909 (< 1.2%)	0/257 (0.0%)
365 days	< 22/2416 (< 0.9%)	< 11/1250 (< 0.9%)	< 11/909 (< 1.2%)	0/257 (0.0%)
Bleeding				
90 days	< 22/2416 (< 0.9%)	< 11/1250 (< 0.9%)	< 11/909 (< 1.2%)	0/257 (0.0%)
365 days	< 22/2416 (< 0.9%)	< 11/1250 (< 0.9%)	< 11/909 (< 1.2%)	0/257 (0.0%)
Neural deficit				
90 days	0/2416 (0.0%)	0/1250 (0.0%)	0/909 (0.0%)	0/257 (0.0%)
365 days	0/2416 (0.0%)	0/1250 (0.0%)	0/909 (0.0%)	0/257 (0.0%)
Vascular injury				
90 days	< 11/2416 (< 0.5%)	< 11/1250 (< 0.9%)	0/909 (0.0%)	0/257 (0.0%)
365 days	< 11/2416 (< 0.5%)	< 11/1250 (< 0.9%)	0/909 (0.0%)	0/257 (0.0%)
Instability				
90 days	< 11/2416 (< 0.5%)	0/1250 (0.0%)	< 11/909 (< 1.2%)	0/257 (0.0%)
365 days	< 33/2416 (< 1.4%)	< 11/1250 (< 0.9%)	< 11/909 (< 1.2%)	< 11/257 (< 4.28%)
Stiffness				
90 days	436/2416 (18.0%)	292/1250 (23.4%)	60/909 (6.6%)	84/257 (32.7%)
365 days	464/2416 (19.2%)	305/1250 (24.4%)	72/909 (7.9%)	87/257 (33.9%)
Periprosthetic fracture				
90 days	< 22/2416 (< 0.9%)	0/1250 (0.0%)	< 11/909 (< 1.2%)	< 11/257 (< 4.28%)
365 days	< 33/2416 (< 1.4%)	< 11/1250 (< 0.9%)	< 11/909 (< 1.2%)	< 11/257 (< 4.28%)
Extensor mechanism disruption				
90 days	< 11/2416 (< 0.5%)	< 11/1250 (< 0.9%)	0/909 (0.0%)	0/257 (0.0%)
365 days	< 11/2416 (< 0.5%)	< 11/1250 (< 0.9%)	0/909 (0.0%)	0/257 (0.0%)
Bearing surface wear				
90 days	0/2416 (0.0%)	0/1250 (0.0%)	0/909 (0.0%)	0/257 (0.0%)
365 days	0/2416 (0.0%)	0/1250 (0.0%)	0/909 (0.0%)	0/257 (0.0%)
Implant loosening				
90 days	< 22/2416 (< 0.9%)	0/1250 (0.0%)	< 11/909 (< 1.2%)	< 11/257 (< 4.28%)
365 days	< 33/2416 (< 1.4%)	< 11/1250 (< 0.9%)	< 11/909 (< 1.2%)	< 11/257 (< 4.28%)
Deep periprosthetic joint infection				
90 days	> 20/2416 (> 0.8%)	< 11/1250 (< 0.9%)	13/909 (1.4%)	0/257 (0.0%)
365 days	> 25/2416 (> 1.0%)	12/1250 (1.0%)	14/909 (1.5%)	< 11/257 (< 4.28%)

**Table 3:** Unadjusted and adjusted odds of adverse events

		**Unadjusted**		**Adjusted**^a^	
**Outcome**	**TJA Group**	**OR (95% CI)**	***P*-value**	**OR (95% CI)**	***P*-value**
**Medical complications**					
90 days	Knee	0.97 (0.76, 1.24)	0.801	0.89 (0.68, 1.18)	0.427
	Shoulder	1.24 (0.85, 1.80)	0.263	0.91 (0.60, 1.37)	0.65
	Hip	REF	REF	REF	REF
365 days					
	Knee	1.08 (0.88, 1.33)	0.438	0.98 (0.78, 1.24)	0.878
	Shoulder	1.66 (1.22, 2.25)	0.001	1.26 (0.90, 1.77)	0.186
	Hip	REF	REF	REF	REF
**Surgical complications**					
90 days	Knee	3.08 (2.38, 3.97)	< 0.001	2.66 (2.03, 3.48)	< 0.001
	Shoulder	4.75 (3.38, 6.68)	< 0.001	4.48 (3.16, 6.35)	< 0.001
	Hip	REF	REF	REF	REF
365 days					
	Knee	2.82 (2.21, 3.60)	< 0.001	2.54 (1.97, 3.27)	< 0.001
	Shoulder	4.36 (3.13, 6.06)	< 0.001	4.10 (2.92, 5.75)	< 0.001
	Hip	REF	REF	REF	REF

^a^Adjusted for age, BMI, readmission score measured at baseline, and average daily MME of opioids within the time window of the endpoint of interest

Surgical complications.

For all-cause surgical complications, the overall rate of complications was 19.9% at 90 days and 21.2% at 365 days. The main driver of surgical complications was stiffness, with a rate of 18.0% at 90 days and 19.2% at 365 days. The highest rates of stiffness at 90 days were in the shoulder (32.7%) and the knee (23.4%) compared to the hip (6.6%). At 365 days, there were only modest increases in stiffness in the shoulder (+ 1.2%), hip (+ 1.3%), and knee (+ 1.0%), indicating that most patients report stiffness in the first 90 days after surgery (Table 2).

For surgical complications, the adjusted odds ratio (aOR) of complications were highest in the knee group (90 days: aOR 2.66, 95% CI 2.03–3.48, 365 days: aOR 2.54, 95% CI 1.97–3.27) and the shoulder group (90 days: aOR 4.48, 95% CI 3.16–6.35, 365 days: aOR 4.10, 95% CI 2.92–5.75) compared to the hip group (Table 3). A comparison of the strength of predictors for events by joint location can be found in Appendix 2.

### Prescriptions filled

Examining group differences in rates of filled prescriptions, the knee group had the highest rates of all prescriptions at 31–365 days (Table 4). In adjusted models, the odds of antiepileptic (*P* < 0.001), NSAIDS (*P* = 0.32), and opioid (*P* < 0.001) prescription are higher in the knee group compared to the hip at 31–90 days. At 0–365 days, the knee group continued to have increased adjusted odds of antiepileptics (*P* = 0.001) and opioids (*P* = 0.005), as well as antibiotics (*P* = 0.032), compared to the hip group, but did not continue to have an increased risk of NSAIDS. However, the shoulder group reported an increase in adjusted odds of antiepileptic (*P* < 0.001) and NSAIDS at 31–90 days (*P* = 0.018), and at 0–365 days there were increased adjusted odds of antiepileptics (*P* = 0.028), but not opioids or NSAIDs (*P* = 0.911) in comparison to the hip group (Table 5).

**Table 4:** Prescription fill rates

	**Overall** ***n* = 2416**	**Knee** ***n* = 1250**	**Hip** ***n* = 909**	**Shoulder** ***n* = 257**
Antibiotic				
31–90 days	465/1733 (26.80%)	256/901 (28.40%)	158/634 (24.90%)	51/198 (25.80%)
0–365 days	1225/1733 (70.70%)	658/901 (73.00%)	431/634 (68.00%)	136/198 (68.70%)
Antiepileptics				
31–90 days	275/1733 (15.90%)	160/901 (17.80%)	70/634 (11.00%)	45/198 (22.70%)
0–365 days	737/1733 (42.50%)	416/901 (46.20%)	232/634 (36.60%)	89/198 (44.90%)
NSAID				
31–90 days	278/1733 (16.00%)	156/901 (17.30%)	85/634 (13.40%)	37/198 (18.70%)
0–365 days	793/1733 (45.80%)	427/901 (47.40%)	283/634 (44.60%)	83/198 (41.90%)
Opioid				
31–90 days	452/1733 (26.10%)	267/901 (29.60%)	136/634 (21.50%)	49/198 (24.70%)
0–365 days	1372/1733 (79.20%)	737/901 (81.80%)	477/634 (75.20%)	158/198 (79.80%)

**Table 5:** Associations between medication filled and TJA group

		**Unadjusted**		**Adjusted**^a^	
	**TJA group**	**OR (95% CI)**	***P*-value**	**OR (95% CI)**	***P*-value**
**Antiepileptic**					
31–90 Days	Knee	1.74 (1.29, 2.35)	< 0.001	1.74 (1.26, 2.40)	< 0.001
	Shoulder	2.37 (1.56, 3.59)	< 0.001	2.42 (1.58, 3.72)	< 0.001
	Hip	REF	REF	REF	REF
0–365 Days	Knee	1.49 (1.21, 1.83)	< 0.001	1.44 (1.16, 1.80)	0.001
	Shoulder	1.41 (1.02, 1.95)	0.035	1.45 (1.04, 2.03)	0.028
	Hip	REF	REF	REF	REF
**Antibiotics**					
31–90 Days	Knee	1.20 (0.95, 1.51)	0.129	1.24 (0.97, 1.57)	0.088
	Shoulder	1.05 (0.72, 1.51)	0.813	0.97 (0.67, 1.41)	0.867
	Hip	REF	REF	REF	REF
0–365 Days	Knee	1.28 (1.02, 1.59)	0.032	1.29 (1.02, 1.63)	0.032
	Shoulder	1.03 (0.73, 1.46)	0.852	0.95 (0.67, 1.35)	0.765
	Hip	REF	REF	REF	REF
**NSAIDS**					
31–90 Days	Knee	1.35 (1.02, 1.80)	0.039	1.39 (1.03, 1.89)	0.032
	Shoulder	1.48 (0.97, 2.27)	0.068	1.70 (1.10, 2.64)	0.018
	Hip	REF	REF	REF	REF
0–365 Days	Knee	1.12 (0.91, 1.37)	0.287	1.07 (0.86, 1.33)	0.531
	Shoulder	0.90 (0.65, 1.24)	0.501	0.98 (0.70, 1.37)	0.911
	Hip	REF	REF	REF	REF
**Opioids**					
31–90 days	Knee	1.54 (1.22, 1.96)	< 0.001	1.68 (1.30, 2.18)	< 0.001
	Shoulder	1.20 (0.83, 1.75)	0.331	1.26 (0.85, 1.86)	0.255
	Hip	REF	REF	REF	REF
0–365 days	Knee	1.48 (1.16, 1.89)	0.002	1.44 (1.11, 1.87)	0.005
	Shoulder	1.30 (0.88, 1.92)	0.188	1.36 (0.91, 2.02)	0.135
	Hip	REF	REF	REF	REF

^a^Adjusted for age, BMI, and readmission score measured at baseline

### Relative costs

In the adjusted total surgical cost model, both the knee and shoulder groups had a significant increase in total costs relative to the hip group, with a 9% and 14% increase in cost, respectively (both *P* = 0.003). Surgical episode costs were also higher in the knee and shoulder groups, with an increase of 6% for TKA and 21% in cost for TSA compared to the hip group (knee: *P* = 0.035; shoulder: *P* < 0.001). In these models, the shoulder group had the highest adjusted odds of increased costs in both total costs (*P* = 0.003) and surgical episode-only costs (*P* < 0.001) (Table 6).

**Table 6:** Association of differences in costs between different TJA groups

		Unadjusted		Adjusted^a^	
Costs^b^	TJA group	Estimate (95% CI)	*P*-value	Estimate (95% CI)	*P*-value
Total health system cost	Knee	1.08 (1.02, 1.15)	0.010	1.09 (1.03, 1.15)	0.003
	Shoulder	1.19 (1.08, 1.32)	< 0.001	1.14 (1.05, 1.25)	0.003
	Hip	REF	REF	REF	REF
Surgical episode cost	Knee	1.06 (1.00, 1.12)	0.050	1.06 (1.00, 1.12)	0.035
	Shoulder	1.21 (1.12, 1.31)	< 0.001	1.21 (1.12, 1.31)	< 0.001
	Hip	REF	REF	REF	REF

^a^Model adjusted for: age, BMI, insurance type, readmission score measured at baseline, and average daily MME of opioid within 1 year of follow-up

^b^Costs adjusted for inflation, 2020 equivalent. Models populated on a cohort of 2367 patients (costs trimmed to 1–99 percentile)

## Discussion

This study analyzed claims data from a cohort of 2416 patients who underwent TKA, THA, or TSA, revealing novel insights into the relative risks of these commonly performed orthopedic procedures. The main findings indicate there is notable variability for adverse events across TJA surgical sites. For example, at 365 days post-surgical, patients undergoing TSA or TKA were at increased odds (aOR = 4.1 and 2.54, respectively) for surgical complications compared to THA (the reference group for these analyses). Those receiving TKA were also at increased odds of having an opioid prescription filled at 31–90 days (aOR = 1.68) and 0–365 days (aOR = 1.44) post-operatively. Furthermore, the TSA and TKA groups were associated with higher total and surgical episode costs when compared to THA. Our findings indicate that patients have an increased risk for surgical complications (both TKA and TSA) and longer-term opioid prescriptions (TKA only) compared to those undergoing THA. Collectively, these results can be used to inform future population-based approaches to managing osteoarthritis care pathways or reimbursement policies for TJA across multiple joint sites. As management of TJA moves towards the expansion of bundled care or disease-based bundling, understanding the relative risk profiles for each TJA location will help determine best practices in patient selection, pre- and post-operative care pathways, and reimbursement patterns.

The incidence of TJA procedures is variable, and the relative numbers seen in this single-center study align with those trends, with shoulder arthroplasty having the lowest incidence [22]. Our cohorts aligned with the rates of adverse events in the literature for hips [23], knees [24], and shoulders [25]. Of note in our study, we included stiffness as a surgical complication. The rates of stiffness reported in each group of the cohort accounted for the majority of the percentage of surgical complications reported. With our TJA site comparison, the shoulder has significantly higher rates of adverse events, including infection, instability, and cardiovascular events. These adverse event rates for TSA should be critically evaluated, given that the volume of TSA is expected to increase. Examining our findings in the context of quality of life (QOL) is speculative because we have no direct measures of QOL. However, these findings do align with literature related to QOL, where, in general, THA patients demonstrate lower rates of complications and higher increases in QOL in comparison to TKA patients [26–29]. There is a paucity of literature to compare changes in QOL in TSA patients relative to TKA and THA, which would be an interesting extension of this analysis, which was focused on administrative data [30]. In general, existing research supports that an increase in QOL is expected following arthroplasty, but further research is needed to examine the relative differences in QOL post-arthroplasty between TKA, THA, and TSA.

In our study, patients undergoing TKA had the highest rates of all prescriptions, including opioids. Studies have reported rates of chronic pain after these procedures ranging from 7–34% [31]. For example, the rate of chronic postoperative pain in TKA patients is 10–34% [32], compared to shoulder (22–28%) and hip patients (7–23%) [33–35]. In a cohort of patients from the same medical center as these analyses, we observed similar rates of post-operative high-impact chronic pain across TKA, THA, and TSA (9–11%) [36]. Traditionally, TKA patients also demonstrate slower improvement in physical function and longer recovery compared to THA patients [37–39]. However, it is beyond the scope of our data to correlate opioid or analgesic prescription rates with chronic postoperative pain for any TJA location.

Lastly, we investigated the difference in the risks of the surgical episode costs and total costs among knee, shoulder, and hip patients. Shoulder patients were at the highest risk for increased costs for both the surgical episode and total costs compared to hip patients. These findings are consistent with the current literature, where TSA has higher, more variable costs compared to THA and TKA [40]. However, it is also noted that in our study, TKA patients also demonstrated higher costs compared to hip patients. These findings, when considered together, provide comparative novel insights for population-based health strategies in managing osteoarthritis with arthroplasty. Traditionally, target pricing for procedural bundles is determined by the payor and focuses on 90-day episodes of care, however, single-payer healthcare systems are contained at the country level, rather than the payor level [41]. Within THA and TKA, implant costs, personnel costs, and, to some extent, medications are the main drivers of costs, regardless of payment model. However, in TSA patients the most significant driver of cost is implant cost and medications [40], and cost and length of stay (LOS) are related to surgical volume [42], whereas in THA and TKA there is conflicting evidence to suggest that surgical volume lowers costs and costs may vary by inpatient versus outpatient surgeries [43–45].

Our study has numerous strengths. Firstly, although our study was from a single health center, we utilized comprehensive data sources that captured all healthcare utilization and costs for patients, and our findings are representative of the current literature but may have limited applicability in countries that do not participate in fee-for-service healthcare. Yet, even in countries with different payment systems, these data could help gain a better understanding of the relative costs of TJA across hip, knee, and shoulder and how resources should be allocated to support the surgical management of OA. Second, our study is the first we are aware of to directly compare outcomes of adverse events, medications, and relative costs across THA, TKA, and TSA. Traditionally, the rates of adverse events and costs are reported by the individual joint or, in other cases, by combining THA and TKA. This combined approach allows for a direct comparison of outcomes across sites to provide novel insights into a broader, population-based approach to managing TJA. Lastly, we reported outcomes of up to 365 days, whereas many comparative studies only report outcomes such as adverse events and costs up to 90 days, as it relates to follow-up times aligned with existing bundled care programs.

This study also has some limitations that should be considered when interpreting these results. Firstly, our study design was a retrospective analysis of claims data; therefore, causal inferences could not be made about the outcomes and specific indications for surgery. Ideally, a study of this magnitude within one health system would be conducted prospectively to enable the collection of additional measures, alternative therapies, or medications not captured in claims data. Additionally, other surgical-level factors such as surgeon experience or implant type would help further evaluate the findings. Secondly, we did not have any patient-level data on clinically relevant outcomes, including patient-reported outcomes, health-related quality of life, functional performance measures, and/or satisfaction with TJA. Lastly, we were only able to report on relative costs and did not allow for reporting on absolute costs. Therefore, these data cannot be used to directly compare costs with these same procedures at other institutions or to other competing procedures that may be of interest in population health management approaches for severe osteoarthritis.

## Conclusions

Our findings highlight areas for further exploration if health systems or payors want to consider value-based care programs for TJA collectively (i.e., THA, TKA, and TSA together). For example, understanding the rates of adverse events and related costs in these populations would inform how to balance risk and manage this population. These analyses are an important precursor to future work that will help to better identify drivers of cost across TJA locations and determine areas for improvement in costs, such as negotiating lower implant costs and streamlining personnel. Finally, estimating pre-operative risk has become considered the standard of care for operative readiness for elective THA and TKA. Our data indicates that parallel efforts in risk estimation for adverse events should be a high priority for TSA, given the observed increased risk of surgical complications. Otherwise, there is a chance that an increasing volume of TSA could be associated with higher adverse event rates than have been observed for TKA and THA.

## Supplementary Information

Supplementary Material 1.

Supplementary Material 2.

## Data Availability

The datasets generated and/or analyzed during the current study are not publicly available due to institutional data-sharing policies.

## Abbreviations

TJA: Total Joint Arthroplasty
THA: Total hip arthroplasty
TKA: Total knee arthroplasty
TSA: Total shoulder arthroplasty
CCJR: Comprehensive Care for Joint Replacement
STROBE: Strengthening the Reporting of Observational Studies in Epidemiology
EHR: Electronic health record
ECI: Elixhauser Comorbidity Index
NSAIDs: Non-steroidal anti-inflammatories
MME: Opioid Morphine Milligram Equivalent
OR: Odds ratios
aOR: Adjusted odds ratios
