# Supplementary material for: Comparison of adverse events, prescription medication, and costs after hip, knee, and shoulder total joint arthroplasty: a retrospective cohort study
Source: Arthroplasty. 2025 May 6;7:24. doi: 10.1186/s42836-025-00309-y (PMC12054235; doi:10.1186/s42836-025-00309-y)
Supplement: Supplementary file 1 — Supplementary Material 1. [file 42836_2025_309_MOESM1_ESM.docx]

Appendix 1: ICD-10 Codes used to classify hip, knee and shoulder medical and surgical complications

|  | ICD-10 Codes |
| --- | --- |
| **Medical Complications** |  |
| Acute renal failure | N17.9, N17.0, N19, N17.8, N99.0, N17.1 |
| Heart Failure | I50.21, I50.9, I50.31, I50.82, I50.20, I50.30, I11.0, I50.810, I50.89, I50.84, I97.131 |
| Myocardial Infarction | I21.9, I22.2, I21.3, I21.A1, I21.4 |
| Osteolysis | T84.050A, T84.051A, T84.052A, T84.053A, T84.058A, T84.059A |
| Respiratory Failure | J96.02, J95.821, J96.01, J96.00, J96.90, J96.91 |
| Stroke | I63.9, I63.132, I63.442, I63.02, I63.40, I63.10, I63.441, I63.12, I63.8, I63.89, I63.032, I63.449, I63.019, I63.30, I63.09, I63.039, I63.49 |
| Thromboembolic disease | I82.402, I82.403, I82.409, I82.411, I82.412, I82.413, I82.419, I82.431, I82.432, I82.433, I82.439, I82.441, I82.442, I82.443, I82.449, I82.491, I82.492, I82.493, I82.499, I82.4Y1, I82.4Y2, I82.4Y3, I82.4Y9, I82.4Z1, I82.4Z2, I82.4Z3, I82.4Z9, I26.01, I26.02, I26.09, I26.90, I26.92, I26.93, I26.94, I26.99, I82.401 |
| Urinary tract infection | N39.0 |
| **Surgical Complications** |  |
| Bearing Surface Wear | T84.060A, T84.061A, T84.062A, T84.063A, T84.068A, T84.069A |
| Bleeding | M96.810,M96.830 |
| Deep periprosthetic joint infection | T84.50XA, T84.51XA, T84.52XA, T845.3XA, T84.54XA, T84.59XA |
| Extensor Mechanism Disruption | M66.251, M66.252, M66.259, M66.261, M66.262, M66.269 |
| Implant fracture or insert dissociation | T84.010A, T84.011A, T84.012A, T84.013A, T84.018A, T84.019A |
| Implant loosening | T84.030A, T84.031A, T84.032A, T84.033A, T84.038A, T84.039A |
| Instability | S83.411A, S83.412A, S83.419A, T84.020A, T84.021A,T84.022A, T84.023A, T84.028A, T84.029A |
| Neural Deficit | G97.82 |
| Periprosthetic fracture | M97.01XA, M97.02XA, M97.11XA, M97.12XA, M97.31XA, M97.32XA, M97.9XXA |
| Stiffness | M25.661, M25.662, M25.669, M25.651, M25.652, M25.659, M25.611, M25.612, M25.619 |
| Vascular Injury | T81.718A, T81.719A, T81.72XA |
| Wound Complication | T81.31XA |

*ICD- 9 codes were converted to ICD-10 codes in this table
