# Supplementary material for: Comparison of adverse events, prescription medication, and costs after hip, knee, and shoulder total joint arthroplasty: a retrospective cohort study
Source: Arthroplasty. 2025 May 6;7:24. doi: 10.1186/s42836-025-00309-y (PMC12054235; doi:10.1186/s42836-025-00309-y)
Supplement: Supplementary file 2 — Supplementary Material 2. [file 42836_2025_309_MOESM2_ESM.docx]

Appendix 2: The effect of specific predictors for each outcome by TJA group

|  | | **Knee** | | | **Hip** | | | **Shoulder** | | |
| --- | --- | --- | --- | --- | --- | --- | --- | --- | --- | --- |
| **Endpoint** | **Predictor** | **OR (95% CI)** | **p-value** | **McFadden’s R2** | **OR (95% CI)** | **p-value** | **McFadden’s R2** | **OR (95% CI)** | **p-value** | **McFadden’s R2** |
|  |  |  |  |  |  |  |  |  |  |  |
| Medical Complications 90 Days | Age (1 yr increase) | 1.06 (1.03, 1.09) | <0.001 | 0.112 | 1.04 (1.02, 1.07) | 0.001 | 0.132 | 1.03 (0.98, 1.09) | 0.221 | 0.103 |
|  | BMI (1 unit increase) | 1.02 (0.99, 1.06) | 0.191 |  | 1.01 (0.97, 1.04) | 0.759 |  | 1.03 (0.98, 1.09) | 0.227 |  |
|  | Readmission score (1 unit increase) | 1.04 (1.03, 1.05) | <0.001 |  | 1.04 (1.03, 1.05) | <0.001 |  | 1.03 (1.02, 1.05) | <0.001 |  |
|  | Race: White vs Other | 1.15 (0.75, 1.76) | 0.522 |  | 0.77 (0.47, 1.27) | 0.306 |  | 0.97 (0.37, 2.54) | 0.950 |  |
|  | Sex: Female vs Male | 1.42 (0.98, 2.05) | 0.064 |  | 1.34 (0.88, 2.03) | 0.167 |  | 0.54 (0.27, 1.09) | 0.088 |  |
|  |  |  |  |  |  |  |  |  |  |  |
|  |  |  |  |  |  |  |  |  |  |  |
| Medical Complications 365 Days | Age (1 yr increase) | 1.06 (1.04, 1.08) | <0.001 | 0.109 | 1.04 (1.02, 1.06) | <0.001 | 0.160 | 1.05 (1.01, 1.10) | 0.015 | 0.149 |
|  | BMI (1 unit increase) | 1.01 (0.99, 1.04) | 0.291 |  | 1.01 (0.97, 1.04) | 0.670 |  | 1.03 (0.98, 1.08) | 0.217 |  |
|  | Readmission score (1 unit increase) | 1.04 (1.04, 1.05) | <0.001 |  | 1.05 (1.04, 1.06) | <0.001 |  | 1.04 (1.02, 1.06) | <0.001 |  |
|  | Race: White vs Other | 1.06 (0.75, 1.50) | 0.726 |  | 1.00 (0.63, 1.59) | 0.986 |  | 0.96 (0.42, 2.23) | 0.927 |  |
|  | Sex: Female vs Male | 1.25 (0.93, 1.68) | 0.143 |  | 1.48 (1.03, 2.12) | 0.033 |  | 0.97 (0.53, 1.75) | 0.911 |  |
|  |  |  |  |  |  |  |  |  |  |  |
| Surgical Complications 30 Days | Age (1 yr increase) | 1.03 (1.01, 1.05) | 0.014 | 0.008 | 1.12 (1.07, 1.17) | <0.001 | 0.098 | 1.00 (0.97, 1.03) | 0.966 | 0.019 |
|  | BMI (1 unit increase) | 1.00 (0.97, 1.03) | 0.851 |  | 1.04 (0.99, 1.10) | 0.110 |  | 0.98 (0.94, 1.02) | 0.339 |  |
|  | Readmission score (1 unit increase) | 1.00 (0.99, 1.01) | 0.341 |  | 1.00 (0.99, 1.02) | 0.803 |  | 0.99 (0.98, 1.01) | 0.392 |  |
|  | Race: White vs Other | 1.04 (0.72, 1.50) | 0.820 |  | 0.92 (0.44, 1.95) | 0.833 |  | 1.12 (0.50, 2.51) | 0.780 |  |
|  | Sex: Female vs Male | 0.99 (0.73, 1.33) | 0.923 |  | 1.53 (0.86, 2.74) | 0.150 |  | 0.87 (0.50, 1.52) | 0.618 |  |
|  |  |  |  |  |  |  |  |  |  |  |
| Surgical Compliations 365 Days | Age (1 yr increase) | 1.03 (1.01, 1.05) | 0.008 | 0.009 | 1.07 (1.04, 1.11) | <0.001 | 0.070 | 1.00 (0.97, 1.04) | 0.836 | 0.025 |
|  | BMI (1 unit increase) | 1.00 (0.98, 1.03) | 0.926 |  | 0.99 (0.95, 1.04) | 0.756 |  | 0.99 (0.95, 1.03) | 0.579 |  |
|  | Readmission score (1 unit increase) | 1.00 (0.99, 1.01) | 0.523 |  | 1.01 (1.00, 1.02) | 0.113 |  | 1.00 (0.98, 1.01) | 0.657 |  |
|  | Race: White vs Other | 0.91 (0.67, 1.24) | 0.554 |  | 1.16 (0.63, 2.15) | 0.630 |  | 1.18 (0.54, 2.60) | 0.675 |  |
|  | Sex: Female vs Male | 1.25 (0.95, 1.64) | 0.107 |  | 1.60 (1.01, 2.54) | 0.043 |  | 1.19 (0.70, 2.02) | 0.528 |  |
